# Microbial Profile of the *Ventriculum* of Honey Bee (*Apis mellifera ligustica* Spinola, 1806) Fed with Veterinary Drugs, Dietary Supplements and Non-Protein Amino Acids

**DOI:** 10.3390/vetsci7020076

**Published:** 2020-06-06

**Authors:** Giovanni Cilia, Filippo Fratini, Elena Tafi, Barbara Turchi, Simone Mancini, Simona Sagona, Antonio Nanetti, Domenico Cerri, Antonio Felicioli

**Affiliations:** 1 Department of Veterinary Sciences, University of Pisa, Viale delle Piagge 2, 56124 Pisa, Italy; filippo.fratini@unipi.it (F.F.); elenat93@libero.it (E.T.); barbara.turchi@unipi.it (B.T.); simone.mancini@unipi.it (S.M.); simonasagona@tiscali.it (S.S.); domenico.cerri@unipi.it (D.C.); antonio.felicioli@unipi.it (A.F.); 2 Interdepartmental Research Center “Nutraceuticals and Food for Health”, University of Pisa, Via del Borghetto 80, 56124 Pisa, Italy; 3 Department of Science, University of Basilicata, via dell’Ateneo Lucano 10, 85100 Potenza, Italy; 4 Department of Pharmacy, University of Pisa, via Bonanno 6, 56126 Pisa, Italy; 5 CREA Research Centre for Agriculture and Environment, Via di Saliceto 80, 40128 Bologna, Italy; antonio.nanetti@crea.gov.it

**Keywords:** GABA, beta-alanine, oxalic acid, diet effect, microbiota

## Abstract

The effects of veterinary drugs, dietary supplements and non-protein amino acids on the European honey bee (*Apis mellifera ligustica* Spinola, 1806) *ventriculum* microbial profile were investigated. Total viable aerobic bacteria, Enterobacteriaceae, staphylococci, *Escherichia coli*, lactic acid bacteria, *Pseudomonas* spp., aerobic bacterial endospores and *Enterococcus* spp. were determined using a culture-based method. Two veterinary drugs (Varromed^®^ and Api-Bioxal^®^), two commercial dietary supplements (ApiHerb^®^ and ApiGo^®^) and two non-protein amino acids (GABA and beta-alanine) were administered for one week to honey bee foragers reared in laboratory cages. After one week, *E. coli* and *Staphylococcus* spp. were significantly affected by the veterinary drugs (*p* < 0.001). Furthermore, dietary supplements and non-protein amino acids induced significant changes in *Staphylococcus* spp., *E. coli* and *Pseudomonas* spp. (*p* < 0.001). In conclusion, the results of this investigation showed that the administration of the veterinary drugs, dietary supplements and non-protein amino acids tested, affected the *ventriculum* microbiological profile of *Apis mellifera ligustica*.

## 1. Introduction

A decline in the honey bee population is threatening both pollination service and the beekeeping industry globally [1,2]. Honey bee colony losses are related to several causes, including habitat modifications, the massive use of agrochemicals, bacterial and parasitic diseases, climate changes, and multifactorial interactions [2,3,4]. 

A key role of gut microorganisms in animal health and welfare has been documented not only in mammals but also in insects [5,6]. The literature provides evidence that the midgut microbiota of eusocial bees, namely honey bees and bumblebees, plays an important role in protecting adults against pathogens [7,8,9,10,11].

The honey bee midgut microbial community was investigated by using culture-dependent methods, resulting in the identification of several microorganisms, including Gram-variable pleomorphic bacteria, *Bacillus* spp. and Enterobacteriaceae, together with moulds and yeasts [12,13,14]. Molecular techniques allowed to repeatedly detect a characteristic gut microbiota of honey bee foragers consisting of nine distinct bacterial phylotypes accounting for the 95% of the total bacterial community [10,15].

The widespread mite *Varroa destructor* (Anderson and Trueman, 2000) and its associated viruses are generally considered among the primary causes of colony collapse [16]. Treatments with acaricides must be administered regularly to reduce the infestation impact on the colony survival. Oxalic and formic acid are natural active compounds commonly used against this mite [17,18,19]. However, when the acids are accidentally ingested, they are likely to alter the intestinal pH and microbial balance [20].

As for bee products, including beeswax [21], the interest in food supplements like vegetable extracts, microorganisms and vitamins is increasing due to their possible role in promoting colony health, vitality and productivity. Anyhow, these types of products may cause modifications of honey bees midgut microbiota [22].

Adult honey bees use nectar as a primary alimentary source, containing sugars and amino acids that could play a major role in the microbiota modifications [23,24]. Gamma-aminobutyric acid (GABA) and beta-alanine are two of the most frequently and abundant non-protein amino acids of nectar [25,26]. These non-protein amino acids received attention for their potential role in mason bee physiology [27,28].

In light of the increasing use of dietary supplements and veterinary drugs in apiculture, the aim of this study was to investigate the modifications in the viable intestinal microbial community of the worker honey bee fed with (*i*) commercial veterinary drugs containing oxalic acid and formic acid, (*ii*) commercial dietary supplements containing yeasts and vitamins or herbal natural extracts and (*iii*) non-protein amino acids, GABA and beta-alanine.

## 2. Materials and Methods

### 2.1. Experimental Design

Seven hundred *Apis mellifera ligustica* (Spinola, 1806) foragers were randomly collected in June 2018 from the same colony in the experimental apiary of the Department of Veterinary Sciences of the University of Pisa (San Piero a Grado, Pisa, Italy). Twenty seven poly(methyl methacrylate) sterilized cages (11 × 13 × 6.5 cm), each including a gravity feeder, one small wax comb, and two transparent walls, were used to host a pool of 25 bees each. The cages were kept between 31 °C and 34 °C with a relative humidity ranging from 50% to 80% until the end of the experiment (one week) [29]. Twenty-five specimens were processed immediately after the sampling and were used as Time 0 control (T0). The remaining honey bees, divided in pool of 25 specimens and reared in cages, were fed with eight different experimental diets and one control diet (C, sterile 1:1 sucrose:water, w:w). Each diet was administrated to three replicate cages ad libitum. Two registered antivarroa veterinary drugs were used: Api-Bioxal^®^ (AB, Chemicals Life, Vigonza, Italy) containing 44.03 g/L of oxalic acid and VarroMed^®^ (VM, BeeVital GmbH, Obertrum am See, Austria) containing 31.42 g/L of oxalic acid and 5 g/L of formic acid. Two commercial dietary supplements containing maltodextrin, yeasts and vitamins (AG, ApiGo^®^, Chemicals Life, Vigonza, Italy) and essential oils, B vitamins as well as the natural extracts of garlic and cinnamon (AH, ApiHerb^®^, Chemicals Life, Vigonza, Italy) were also employed. Furthermore, two non-protein amino acids were used at two different concentrations (GABA 1×, GABA 0.75 mM; GABA 20×, GABA 15 mM; BALA 1×, beta-alanine 2.3 mM; BALA 20×, beta-alanine 46 mM).

Diets AB, VM, AG, and AH were formulated according to the producers’ instructions. Non-protein amino acids (GABA 1x, GABA 20x, BALA 1x and BALA20x) were mixed with sterile 1:1 sucrose:water (w:w) to reach the aforementioned concentrations. At the beginning of the trial (T0) and after one week (Time7, T7), in order to induce hypothermia and avoid the intestinal microbial profile modification, the honey bees were frozen at −20 °C for 5 min and washed into 95% ethanol to remove the external microbial contamination, right after the *ventriculum* (small intestine and midgut) were dissected for the microbiological analyses. At T7, no mortality was observed in the reared honey bees.

### 2.2. Microbiological Analyses

The *ventriculi* of the 25 bees belonging to a single cage were pooled (three replicate pools for each diet) and suspended into 1 mL of sterile phosphate buffered saline (PBS) solution. The samples were homogenized for 60 s with a Stomacher^®^ 400 Circulator (VWR International Srl, Milan, Italy). Ten-fold serial dilution series were performed, and different media were used in order to quantify the different target microorganisms. The total viable aerobic count was enumerated on Plate Count Agar (PCA) after incubation at 30 °C for 72 h; the Enterobacteriaceae were quantified on violet red bile glucose agar (VRBGA) after incubation at 37 °C for 24 h; the staphylococci were enumerated on mannitol salt agar (MSA) after incubation at 37 °C for 24–48 h; *Escherichia coli* were enumerated on a tryptone bile X-glucuronide medium (TBX) after incubation at 42 °C for 24 h; de Man–Rogosa–Sharpe agar (MRS) was employed to enumerate the lactic acid bacteria after incubation at 37 °C for 48 h in anaerobic conditions; *Pseudomonas* spp. were quantified on a penicillin pimaricin agar (PPA) plate incubated at 30 °C for 24–48 h; *Enterococcus* spp. were evaluated on kanamycin aesculin azide agar (KAAA) after incubation at 42 °C for 24 h; and the aerobic bacterial endospores were enumerated on PCA (incubation at 37 °C for 48), after a heat-shocking at 80 °C for 10 min of the dilutions. All the culture media and supplements were purchased by ThermoFisher Scientific (Milan, Italy). The bacterial counts were expressed as log Colony-Forming Unit (CFU) per samples.

### 2.3. Statistical Analysis

Variations that occurred in microbial loads between T0 and T7 were tested by Student’s t-tests. Statistical differences between the control groups and the treated ones at T7 were tested by one-way ANOVA. If a significant variation was detected a Tukey’s test was performed as post hoc multiple comparisons. The effects were considered significant when *p* < 0.05. Statistical analyses were performed with R free software [30].

## 3. Results

### 3.1. Effects of VarroMed^®^ and Api-Bioxal^®^ on the Viable Honey Bee Ventriculum Microbial Profile

The evaluation of the *ventriculum* microbial profile of bees fed with C diet and diets with VM and AB is summarized in Table 1. The bacterial endospores were absent in all the samples; thus, they were not reported.

After one week of experimental diets, both the total viable aerobic counts and Enterobacteriaceae counts increased (both at *p* < 0.001 for all the diets), with no statistical differences within the treatments (*p* = 0.798 and *p* = 0.455 for viable aerobic and Enterobacteriaceae counts, respectively). The *Staphylococcus* spp. and *E. coli* amounts were not affected after one week of the C diet (*p* = 0.932 and *p* = 0.887, respectively), on the contrary, both the tested veterinary drugs statistically influenced both parameters (*p* = 0.019 and *p* < 0.001 for *Staphylococcus* spp. and *p* = 0.016 and *p* = 0.022 for *Escherichia coli,* for, respectively, VM and AB). VarroMed^®^ completely inhibited *E. coli* and *Staphylococcus* spp., on the other hand, Api-Bioxal^®^ doubled the *E. coli* and *Staphylococcus* spp. loads. After one week of treatment, for all the samples, modifications of *E. coli* (*p* < 0.001) and *Staphylococcus* spp. (*p* < 0.001) counts were statistically affected with higher counts in bees fed with AB, followed by those fed the control diet and VM, respectively.

Statistical difference was found between T0 and AB for *Pseudomonas* spp. determination (*p* = 0.002). After one week, the bees fed with AB showed a higher load of these bacteria than at T0. No statistical differences were detected for *Pseudomonas* spp. counts after one week of the control diet and AB compared to VM, nevertheless, the AB samples showed higher amounts than those from the C group (*p* = 0.013).

After one week, both the veterinary drugs induced a significant increase in *Enterococcus* spp. (*p* < 0.001). Anyhow, due to the high variations among the data, no statistical differences were observed between treatments at T7 (*p* = 0.237).

Compared to T0, honey bees treated with VM for one week showed a significant increase in lactic acid bacteria counts (*p* = 0.003). At T7, VM showed the highest values, while AB showed the lowest; moreover, the C diet was statistically comparable to the two diets including veterinary drugs (*p* = 0.023).

### 3.2. Effects of ApiGo^®^ and ApiHerb^®^ on the Viable Honey Bee Ventriculum Microbial Profile

The microbiological effect of AG and AH on honey bees *ventriculum* microbial profile is reported in Table 2, except for the bacterial endospores which were never detected.

After one week, the total viable aerobic counts increased in both C and the two diets including the dietary supplements AG and AH compared to T0 (*p* < 0.001, *p* = 0.008 and *p* = 0.012, respectively, for C, AG and AH) with no statistical differences between the diets (*p* = 0.328).

After one week, the Enterobacteriaceae significantly increased in both C and AG compared to T0 (*p* < 0.001 and *p* = 0.009, respectively), while AH did not differ from T0 (*p* = 0.703). Consequently, Enterobacteriaceae were significantly higher in both C and AG than in AH (*p* = 0.004). Statistical differences were highlighted for *E. coli* (*p* = 0.016) and *Pseudomonas* spp. (*p* = 0.002) only between the T0 and AH treatments. Bees fed with AH showed a significantly lower amount of both *E. coli* (*p* = 0.001) and *Pseudomonas* spp. (*p* < 0.001) than those fed with both C and AG. After AH administration, *Escherichia coli* and *Pseudomonas* spp. were not detected. 

*Staphylococcus* spp. was not detected at T0, and was found only in honey bees fed with AG for one week (*p* = 0.012). Statistical differences were found for lactic acid bacteria, only between the T0 and AG treatment (*p* = 0.012). Indeed, the AG induced a significant increase in lactic acid bacteria. Among the dietary supplement, only the AG differed significantly compared to T0.

After one week, both dietary supplements, as well as the C diet, induced an increase in *Enterococcus* spp. compared to T0 (*p* = 0.009, *p* = 0.001 and *p* < 0.001 for C, AG and AH, respectively). No statistical differences were found among the diets (*p* = 0.250).

### 3.3. Effects of GABA and Beta-Alanine on the Viable Honey Bee Ventriculum Microbial Profile

The results of the non-protein amino acids on the honey bee *ventriculum* microbial profile are reported in Table 3.

After one week, the total viable aerobic counts increased in C and all the non-protein amino acid-enriched diets, except for GABA 20x (*p* < 0.001, *p* = 0.002, *p* = 0.003, *p* = 0.001 and *p* = 0.017, respectively, for C, β-alanine, β-alanine 20x, GABA and GABA 20x). No statistical differences were found among the diets after one week (*p* = 0.793). 

After one week, Enterobacteriaceae significantly increased in both C and all the non-protein amino acid-enriched diets (*p* < 0.001, *p* = 0.007, *p* = 0.001, *p* = 0.009 and *p* = 0.001, respectively, for C, β-alanine, β-alanine 20x, GABA and GABA 20x). No statistical differences were evidenced among the diets (*p* = 0.787).

After one week of all the non-protein amino acid-enriched diets administration, *Staphylococcus* spp. significantly decreased compared to T0 (*p* < 0.001 for all the diets), while, after one week of the C diet administration, *Staphylococcus* spp. was not detected (*p* = 0.932).

Statistical differences were found for *E. coli* between T0 and β-alanine 20x and GABA (*p* = 0.014 and *p* = 0.016, respectively). In both GABA diets, *E. coli* abruptly decreased, while in β-alanine 20x they significantly increased. 

Compared to T0, *Pseudomonas* spp. was not detected in the bees fed with non-protein amino acid-enriched diets (*p* = 0.002 for all investigated non-proteins amino acids diets), while it was present after one week of the C diet administration (*p* = 0.579).

No statistical differences were found for lactic acid bacteria among the T0 and all the experimental diets (*p* = 0.067, *p* = 0.208, *p* = 0.013, *p* = 0.029 and *p* = 0.028, respectively, for C, β-alanine, β-alanine 20x, GABA and GABA 20x). Likewise, no statistical differences were found among C and non-protein amino acids for lactic acid bacteria (*p* = 0.503). No statistical differences were found between T0 and C, β-alanine and β-alanine 20x for *Enterococcus* spp. (*p* = 0.098, *p* = 0.666 and *p* = 0.244, respectively). Among the non-protein amino acid-enriched diets, GABA and GABA 20x differed significantly from the T0 (*p* = 0.001 and *p* = 0.044, respectively). Bacterial endospores were never detected in the honey bees treated with non-protein amino acids.

## 4. Discussion

Concerning the veterinary drugs, Api-Bioxal is composed of oxalic acid, while VarroMed of both oxalic and formic acid, in concentrations of 44 mg/mL and 5 mg/mL, respectively. Oxalic acid usually shows an antibacterial activity [31], as well as formic acid which was reported as a powerful antimicrobial agent even at low concentrations [32,33,34]. Notably, it seems that formic acid or its synergistic activity with oxalic acid could play a role in the antimicrobial activity of VM. As reported by Raftari et al. (2009), formic acid showed antibacterial activity in vitro against *E. coli* and *S. aureus* [35]. Few research studies reported the *Pseudomonas* spp. tolerance, production and metabolic activity in relation to oxalic acid. Nagarajkumar et al. (2005) reported the ability of several strains of *P. fluorescens* to detoxify soil from oxalic acid produced by fungi, associated to the presence of plasmid genes [36]. Hamel et al. (1999) highlighted the production of oxalic acid as a response to aluminum stress in *P. fluorescens* [37]. As reported in this investigation, the production of oxalic acid in the response to stress suggests that *Pseudomonas* spp. could use it as a substrate. As reported before for *Pseudomonas* spp., *Enterococcus* species could also degrade oxalic acid through metabolic pathways [38]. Even if an antimicrobial activity against *Enterococcus* spp. is reported for formic acid [39,40], probably the combination with oxalic acid in VM may modify or reduce the effect of formic acid. The growth of some bacteria is probably due to their ability to use organic acids as a nitrogen and energy source [41,42]. Oxalic acid, the main component of Api-Bioxal^®^, showed and antibacterial effect against different strains of *Lactobacillus* spp. [43]. Thus, normal miticide treatments may negatively affect honey bees, since many *Lactobacillus* species are linked to honey bee health, inhibiting some potential bacterial pathogens [44,45]. VM is composed of both oxalic and formic acids, and it seemed that *E. coli* was not inhibited by oxalic acid used alone, while it was strongly inhibited by VM treatment.

Concerning the dietary supplement, AH is composed of garlic, cinnamon, mint and thyme which exert an antimicrobial activity against several bacterial strains. The antibacterial activity of garlic against *E.coli*, Pseudomonadaceae and Enterobacteriaceae was widely discussed in literature [46,47,48] and attributed to the presence of mono, di, tri and tetra diallyl sulphides which increase with the number of sulphur atoms in the compound [49,50]. Peppermint showed antibacterial activity against several pathogens, including *E. coli* and *Pseudomonas* spp. [51,52]. Its antibacterial activity is mainly due to terpens, namely α-pinene, limonene and α-terpineol [53]. α-pinene, limonene and α-terpineol, together with thymol and linalool [54] which contributed to the antibacterial activity of *Thymus* against *E. coli* and *Pseudomonas* spp. as well [54,55], were reported in this investigation.

Moreover, the antibacterial activity of AH observed in this study could be due to the antibacterial compounds present in cinnamon, as well as cinnamaldehyde, eugenol and cinnamyl acetate, followed by other terpens (including α-pinene, limonene, α-terpineol and linalool) [56]. *Cinnamomun zeylanicum* showed antibacterial effects against many bacteria, such as *E. coli* and *Pseudomonas* spp. [57,58,59], as highlighted in this investigation with the AH diet.

The garlic and cinnamon present in AH showed an antimicrobial activity against *Staphylococcus* spp. The antibacterial activity of garlic extract has been demonstrated against *S. aureus,* as well as methicillin-resistant and streptomycin-resistant strains, and *S. epidermidis* [60,61]. The antimicrobial activity of garlic is probably due to the action of allicinlin, thiosulfinates, flavonoids and other phenols [62].

It is noteworthy that maltodextrins, present in AG, were metabolized by lactic acid bacteria and used as an energy source, as highlighted in this investigation [63,64].

Although the anti-*Enterococcus* activity of garlic and cinnamon extracts have been widely discussed [65,66], in this investigation the AH diet did not allow the inhibition of *Enterococcus* spp., but caused their increase. On the other hand, the increase in *Enterococcus* spp. observed after the administration of the AG diet was probably due to the capability of this genus to use maltodextrins as a carbon source, as described for lactic acid bacteria [63].

Finally, the action of non-protein amino acids against microorganisms was partially investigated. In this investigation, the effects of GABA on *E. coli* and *Enterococcus* spp. suggested a possible dose-dependent action. A dose-dependent effect of some nectar secondary compounds, including non-protein amino acids, was recognized in some species of *Bombus* [67].

In plants, GABA accumulates in response to various abiotic and biotic stresses, including fungal and bacterial infections [68]. GABA is suggested to mediate the interactions between plants and microorganisms, including bacteria [68,69]. For instance, the *Brassica rapa* plant extracts showed an increase in its in vitro antibacterial activity against *E. coli*, *Pseudomonas aeruginosa* and *Staphylococcus aureus*, after supplementation with GABA [70]. To the best of authors’ knowledge, previous studies regarding the in vitro antibacterial effect of GABA are not available. The growth of *E. coli* was not negatively affected by GABA at the concentration present in nectar. Although, some *E. coli* strains could be able to use GABA as a source of nitrogen [71,72].

In beta-alanine-enriched diets, as in GABA, *Staphylococcus* spp. and *Pseudomonas* spp. were not detected. Moreover, a diet enriched with beta-alanine at both concentrations increased the number of *E. coli* colonies. In the biosynthesis of pantothenic acid in *E. coli,* beta-alanine served as a direct precursor [73,74]. Probably, the presence of ready-to-use beta-alanine could promote the growth of *E. coli.* This growth is significantly higher when beta-alanine is administered 20× compared to lower concentrations, suggesting a dose-dependent effect. At the best of authors’ knowledge, studies on the in vitro antibacterial effect of beta-alanine are not present in the literature, while there are some on the antibacterial activity of beta-alanine-synthetized derivates [75,76].

## 5. Conclusions

In conclusion, the reported effects on the microbiological profile of honey bee *ventriculum* may be direct (killing bacteria and inhibiting its growth, etc.) or indirect (improving the bee immunity system, favoring the development of microbiota able to prevent pathogen strain colonization, etc.). All these factors must be considered in the framework of an increasing interest towards the formulation of dietary supplements for honey bee nutrition. In light of the obtained results, non-protein amino acid-enriched nutrition could be used in order to mitigate the possible beneficial microflora imbalance after anti-Varroa and anti-nosemosis treatments.

## Figures and Tables

**Table 1:** Bacterial counts (log Colony-Forming Unit (CFU) per samples ± s.d.) obtained from honey bees’ *ventriculum* after one week of veterinary drugs administration.

Bacteria	Time 0	Diet (Veterinary Drugs and Control) One Week			*p*-Value §
		C	VM	AB	
Total viable aerobic counts	5.36 ± 0.51	7.28 ± 0.71 *	7.03 ± 0.88 *	7.40 ± 0.11 *	0.798
Enterobacteriaceae	3.34 ± 0.77	5.88 ± 0.89 *	5.18 ± 0.04 *	5.59 ± 0.75 *	0.455
*Escherichia coli*	2.43 ± 1.28	2.52 ± 0.95 ^b^	0.00 ± 0.00 *^,c^	4.72 ± 0.44 *^,a^	<0.001
*Pseudomonas* spp.	2.69 ± 0.96	2.93 ± 0.32 ^b^	3.36 ± 0.93 ^a,b^	4.38 ± 0.37 *^,a^	0.013
*Staphylococcus* spp.	1.47 ± 0.69	1.50 ± 0.55 ^b^	0.00 ± 0.00 *^,c^	3.48 ± 0.13 *^,a^	<0.001
*Enterococcus* spp.	1.24 ± 0.34	2.45 ± 1.58	3.88 ± 1.04 *	3.81 ± 0.63 *	0.237
Lactic acid bacteria	2.87 ± 0.67	3.91 ± 1.03 ^a,b^	5.26 ± 0.97 *^,b^	2.74 ± 0.05 ^a^	0.023

* Means within row different from the Time (T0) (*p* < 0.05). ^a,b,c^ Means within row lacking a common superscript are different (*p* < 0.05). § *p*-value of ANOVA between the treatments after one week of the experimental diet (Control (C), VarroMed^®^ (VM) and Api-Bioxal^®^ (AB)).

**Table 2:** Bacterial counts (log CFU per samples log CFU per samples ± s.d.) obtained from honey bees’ *ventriculum* after one week of dietary supplement administration.

Bacteria	Time 0	Diet with Dietary Supplements, One Week			*p*-Value ^§^
		C	AG	AH	
Total viable aerobic counts	5.36 ± 0.51	7.28 ± 0.71 *	6.76 ± 0.59 *	6.62 ± 0.56 *	0.328
Enterobacteriaceae	3.34 ± 0.77	5.88 ± 0.89 *^,a^	5.61 ± 1.16 *^,a^	3.16 ± 0.13 ^b^	0.004
*Escherichia coli*	2.43 ± 1.28	2.52 ± 0.95 ^a^	3.20 ± 0.32 ^a^	0.00 ± 0.00 *^,b^	0.001
*Pseudomonas* spp.	2.69 ± 0.96	2.93 ± 0.32 ^a^	3.11 ± 0.49 ^a^	0.00 ± 0.00 *^,b^	<0.001
*Staphylococcus* spp.	1.47 ± 0.69	1.50 ± 0.55 ^b^	3.29 ± 0.03 *^,a^	0.00 ± 0.00 ^c^	<0.001
*Enterococcus* spp.	1.24 ± 0.34	2.45 ± 1.58 *	3.68 ± 1.03 *	3.86 ± 0.42 *	0.250
Lactic acid bacteria	2.87 ± 0.67	3.91 ± 1.03	4.51 ± 0.70 *	3.05 ± 0.28	0.160

* Means within row different from the Time (T0) (*p* < 0.05). ^a,b,c^ Means within row lacking a common superscript are different (*p* < 0.05). ^§^ *p*-value of ANOVA between the treatments after one week of the experimental diet (Control, ApiGo^®^ (AG) and ApiHerb^®^ (AH)).

**Table 3:** Bacterial counts (log CFU per samples log CFU per samples ± s.d.) obtained from the honey bees’ *ventriculum* after one week of non-protein amino acid (GABA and β-alanine)-enriched diet administration.

Bacteria	Time 0	Diet with Non-Protein Amino Acids, One Week					*p*-Value ^§^
		Control	β-Alanine	β-Alanine 20x	GABA	GABA 20x	
Total viable aerobic counts	5.36 ± 0.51	7.28 ± 0.71 *	7.42 ± 0.8 *	6.97 ± 0.47 *	7.26 ± 0.54 *	6.79 ± 0.90 *	0.793
Enterobacteriaceae	3.34 ± 0.77	5.88 ± 0.89 *	5.48 ± 0.86 *	6.12 ± 0.25 *	5.55 ± 1.08 *	6.12 ± 0.50 *	0.787
*Escherichia coli*	2.43 ± 1.28	2.52 ± 0.95 ^b^	3.43 ± 0.94 ^a,b^	5.00 ± 0.55 *^,a^	0.00 ± 0.00 *^,c^	2.73 ± 0.64 ^b^	<0.001
*Pseudomonas* spp.	2.69 ± 0.96	2.93 ± 0.32 ^a^	0.00 ± 0.00 *^,b^	0.00 ± 0.00 *^,b^	0.00 ± 0.00 *^,b^	0.00 ± 0.00 *^,b^	<0.001
*Staphylococcus* spp.	1.47 ± 0.69	1.50 ± 0.55 ^a^	0.00 ± 0.00 *^,b^	0.00 ± 0.00 *^,b^	0.00 ± 0.00 *^,b^	0.00 ± 0.00 *^,b^	<0.001
*Enterococcus* spp.	1.24 ± 0.34	2.45 ± 1.58 ^a^	1.39 ± 0.67 ^a,b^	1.52 ± 0.23 ^a,b^	0.00 ± 0.00 *^,b^	0.73 ± 0.03 *^,a,b^	0.044
Lactic acid bacteria	2.87 ± 0.67	3.91 ± 1.03	3.51 ± 0.59	4.83 ± 1.14 *	4.67 ± 1.36 *	4.39 ± 0.57 *	0.503

* Means within row different from the Time 0 (T0) (*p* < 0.05). ^a,b,c^ Means within row lacking a common superscript are different (*p* < 0.05). ^§^ *p*-value of ANOVA between the treatments after one week of the diet (Control, β-alanine, β-alanine 20x, GABA and GABA 20x).
